# Proteomic Profiling of PTEN Inhibition on Periodontal Ligament Stem Cells

**DOI:** 10.1016/j.identj.2026.109493

**Published:** 2026-03-20

**Authors:** Suphalak Phothichailert, Nunthawan Nowwarote, Chatvadee Kornsuthisopon, Shinya Murakami, Supreda Suphanantachat Srithanyarat, Thanaphum Osathanon

**Affiliations:** a Oral Biology (International Program), Faculty of Dentistry, Chulalongkorn University, Bangkok, Thailand; b Center of Excellence for Dental Stem Cell Biology, Faculty of Dentistry, Chulalongkorn University, Bangkok, Thailand; c Université Paris Cité, INSERM UMR1163, Institut Imagine, Paris, France; d Department of Anatomy, Faculty of Dentistry, Chulalongkorn University, Bangkok, Thailand; e Department of Periodontology and Regenerative Dentistry, Graduate School of Dentistry, The University of Osaka, Osaka, Japan; f Department of Periodontology, Faculty of Dentistry, Chulalongkorn University, Bangkok, Thailand

**Keywords:** PTEN inhibitor, VO-OHpic, Mesenchymal stem cells, Proteomics, MAPK cascade, Periodontal regeneration

## Abstract

**Objective:**

Phosphatase and tensin homolog (PTEN) is a critical regulator of cell proliferation, differentiation, and inflammatory balance. However, its downstream proteomic effects in periodontal ligament stem cells (PDLSCs) remain poorly understood. This study aimed to elucidate the proteomic alterations induced by PTEN inhibition and identify potential molecular pathways underlying periodontal regeneration.

**Materials and Methods:**

PDLSCs were treated with 5 μM VO-OHpic for 24 hours, followed by proteomic profiling using mass spectrometric analysis. The resulting proteomic data were analysed using Heatmapper, Metascape, and WebGestalt (WEB-based Gene Set Analysis Toolkit) databases, as well as Cytoscape, to evaluate protein expression patterns and protein–protein interaction networks.

**Results:**

Proteomic analysis identified 7,525 proteins across all samples, with 3,497 proteins commonly expressed between groups. In the VO-OHpic-treated cells, proteins related to cytoskeletal dynamics (ACTR2, FNDC3A), vesicular transport (GGA1, RAB9A), and signal transduction (TRAF3IP3, GJB3) were upregulated. Enrichment analysis identified potential biological pathways related to membrane trafficking, secretion, and regulation of MAPK and Wnt signalling. Conversely, proteins linked to transcriptional and metabolic regulation (GLIS3, PHGDH, NADSYN1) and membrane trafficking (SNX1, REEP1) were downregulated. Cytokine signalling and interferon pathways were explored as the potential pathways involved in these downregulated proteins.

**Conclusion:**

In conclusion, PTEN inhibition by VO-OHpic was associated with proteomic changes in PDLSCs linked to regenerative phenotype, including the modulation of MAPK and Wnt signalling pathways alongside alterations in immune and metabolic pathways. These findings suggest that PTEN may function as a modulator balancing inflammatory regulation and differentiation processes, providing mechanistic insight into its potential role in periodontal tissue regeneration.

## Introduction

The phosphatase and tensin homolog deleted on chromosome 10 (PTEN) is a tumour suppressor gene located at chromosome 10q23.^1^ The protein encoded by PTEN contains 403 amino acids. The major function of PTEN is binding to membrane lipids in its substrate region. PTEN acts as a dual-specificity phosphatase, dephosphorylating both phospholipids and proteins.^2^ Specifically, it dephosphorylates the lipid substrate phosphatidylinositol-3,4,5-trisphosphate (PIP3) at the 3′ position, converting it into phosphatidylinositol-4,5-bisphosphate (PIP2) at the cytoplasmic membrane, thereby modulating PIP3 levels and regulating key biological signalling pathways.^3^^,^^4^

PTEN has been implicated in several biological processes, including osteogenic differentiation,^5^ stem cell regulation,^6^ and inflammation.^7^ During tooth development, PTEN is expressed in the dental papilla, enamel organ, and cervical loops throughout the phases of proliferation, differentiation, and normal tooth development.^6^ Previous studies have reported that loss of PTEN function results in increased bone turnover in the periodontal tissues, characterised by excessive cementum deposition and significantly increased bone volume surrounding the molars *in vivo*.^8^^,^^9^ These findings suggest that PTEN plays a critical role in maintaining periodontal structure homeostasis and bone remodelling, and in regulating proliferation and osteogenic differentiation.

A previous study demonstrated that PTEN inhibition results in decreased cell proliferation and colony-forming unit (CFU) formation in human dental pulp cells.^10^ Further, PTEN inhibition attenuated mineral deposition in human dental pulp cells and intracellular lipid accumulation in human adipose tissue-derived stromal cells. These data imply that PTEN plays a crucial role in cell proliferation and differentiation. Yet, the regulatory pathway(s) involved in this phenomenon have not been elucidated.

Proteomic analysis is one of the most powerful analytical approaches for investigating the function and structure of proteins within an organism. It provides valuable insights into disease progression and facilitates the evaluation of therapeutic effects.^11^^,^^12^ In a PTEN-knockout mouse model, proteomic analysis has revealed key regulatory molecules, including Akt, NF-κB, and p53, that may serve as potential biomarkers to guide chemoprevention and interception strategies in a clinically relevant model of prostate cancer.^13^ So far, PTEN has not been specifically investigated in proteomic studies of dental stem cells. This gap underscores the importance of examining how PTEN shapes protein expression and signalling networks that govern dental stem cell differentiation, proliferation, and regenerative capacity. Accordingly, the present study aimed to identify potential regulatory pathway(s) associated with PTEN inhibition in human periodontal ligament stem cell proliferation and differentiation using proteomic analysis. In addition, a confirmatory experiment was performed to assess the effects of PTEN inhibition on genes involved in cell proliferation and osteogenic differentiation.

## Materials and methods

### Cell isolation and culture of human periodontal ligament stem cells

Periodontal ligament stem cells (PDLSCs) were isolated from periodontal ligament tissue obtained from third molars extracted from 3 donors (ages 18-44 years), which were surgical removal according to each patient's treatment plan. The protocol was approved by the Human Research Ethics Committee of Chulalongkorn University (approval No. HREC-DCU 2025-006). Briefly, the periodontal ligament tissue was collected, minced, and cultured in tissue culture plates for cell explantation. Cells were maintained in Dulbecco’s Modified Eagle’s Medium (DMEM) (Gibco) supplemented with 10% fetal bovine serum (Gibco, USA), 2 mM L-glutamine (Gibco), and 100 units/mL antibiotic and antimycotic (Gibco, USA). PDLSCs were incubated in a humidified atmosphere with 5% CO_2_ at 37°C, and the culture medium was changed every 2-3 days. The cells were subcultured and used for experiments between passages 2 and 5. The expression of mesenchymal stem cell surface markers was examined using flow cytometry analysis including CD44 FITC (BD Bioscience Pharmingen), CD73 FITC (BD Bioscience Pharmingen), CD105 PE (BD Bioscience Pharmingen), and hematopoietic surface marker CD45 (Immuno Tools), were analyzed using flow cytometry FACSCalibur Flow Cytometer (Becton Dickinson, Worldwide Inc.)., and the differentiation toward osteogenic and adipogenic lineages was determined using Alizarin Red S staining and Oil Red O staining, respectively. The stem cell characters were previously reported.^14^ All proteomic experiments were performed using cells derived from three independent donors. For functional validation experiments (qRT-PCR and mineralization assay), cells from an additional independent donor were included (total n = 4 donors).

### Sample preparation

PDLSCs at passage 3 were seeded at a density of 3 × 10^5^ cells/wells in 6 well-plate and cultured with growth medium, incubated in a humidified atmosphere with 5% CO_2_ at 37°C for 24 h. Cells were treated with 5 μM VO-OHpic (Sigma-Aldrich, United States) in growth medium for 24 h, a concentration previously reported in earlier studies. After treatment, cells were trypsinised, collected by centrifugation at 2,500 rpm, and washed with phosphate-buffered saline (PBS). Pellets were collected in PBS for the subsequent analysis.

### Proteomics analysis and statistical analysis

All proteomic experiments were performed using cells derived from three independent donors. Proteomic analysis was performed using liquid chromatography–tandem mass spectrometry. Mass spectrometry (MS) data were analysed using MaxQuant software (version 1.6.2.10) integrated with the Andromeda search engine for protein identification. The Homo sapiens protein database was obtained from UniProt (www.uniprot.org), and a label-free quantification (LFQ) approach was applied. Parameters were set as follows: oxidation of methionine and acetylation of the N-terminus were configured as variable modifications, whilst carbamidomethylation of cysteine was set as a fixed modification. The Bruker Q-TOF instrument was chosen, with peptide mass tolerances set to 0.5 for the first search and 0.25 for the main search.

Bioinformatics and data analyses were performed using the *Homo sapiens* database with a false discovery rate (FDR) < 0.05 and log2 fold change > 1 or < -1.^15^ Label-free quantification (LFQ) intensities were compared between control and VO-OHpic–treated groups using Student’s *t*-test, and proteins were ranked by *P*-value. The top 50 differentially expressed proteins were visualised using Heatmapper software,^16^ with protein expression levels presented as log₂-transformed normalised ratios. Heatmap values represent row-wise Z-score–normalised expression levels. Venn diagram were generated to illustrate the overlap and number of identified proteins between groups.^17^ For proteins with zero detected expression values across all samples in either the control or treatment group, fold-change calculations could not be performed. These proteins were therefore classified using a binary ‘on/off’ approach. Proteins with undetectable expression in the control group but detectable expression in the treatment group were defined as ‘on’ proteins, whereas proteins with detectable expression in the control group but undetectable expression in the treatment group were classified as ‘off’ proteins. Pathway enrichment analysis of differentially expressed proteins was conducted using WebGestalt (WEB-based Gene Set Analysis Toolkit) based on the Kyoto Encyclopedia of Genes and Genomes (KEGG) database,^18^ with log₂ ratios mapped to indicate up- and downregulated proteins. Protein-protein interaction (PPI) network analysis was performed using Metascape, incorporating GO molecular function, GO biological processes, KEGG pathways, Reactome, and canonical pathway datasets,^19^ and visualised using Cytoscape.^20^ The data on protein expression were shown in Supplementary file 1.

### RNA extraction and gene expression analysis

Cells derived from four independent donors were used for gene expression analysis. Cells were treated with the VO-OHpic (5 μM) at 24 h. Total RNA was extracted using TRIzol reagent (RiboEx, GeneAll® Seoul, Korea) and converted into cDNA using a reverse transcription kit (Promega). The real-time polymerase chain reaction (qRT-PCR) was observed using FastStart SYBR Green Master Essential DNA (Roche Applied Science) with CFX connect Real-Time PCR (Bio-Rad). The cycling conditions were set to 95°C for 30 s, followed by 40 cycles of 95°C for 3 s and 60°C for 30 s. Expression levels were calculated from the quantification cycle (Cq). The reference gene used was *GAPDH*. Melting curve analysis was performed to examine the product specificity.^21^ Relative gene expression was quantified using the comparative Ct method (2^−ΔΔCt^ method).^21^ Oligonucleotide sequences are *TP53 forward:5’*-TGCGTGTGGAGTATTTGGATG-3’, *TP53 reverse: 5’*-TGGTACAGTCAGAGCCAACCTC-3’; *NANOG forward:5’*-ATGCCTCACACGGAGACTGT-3’, *NANOG reverse: 5’*-AAGTGGGTTGTTTGCCTTTG-3’, *GAPDH forward:5’*- CACTGCCAACGTGTCAGTGGTG-3’, *GAPDH reverse: 5’*-GTAGCCCAGGATGCCCTTGAG-3’.

### Mineralization assay

Cells derived from four independent donors were maintained in osteogenic induction medium with or without VO-OHpic (5μM) for 14 days. Cells were then fixed with 4% cold formaldehyde in PBS for 15 min, washed with deionised water, and stained with a 1% Alizarin Red S solution (cat. No. A5533, Sigma-Aldrich, United States) for 5 min.

### Statistical analyses

All statistical analyses were conducted using GraphPad Prism version 9 software (GraphPad Software Inc.). Data were analysed using the Mann-Whitney test for 2-group comparisons. The statistically significant difference was considered when *P* < .05.

## Results

### Characteristics of cells isolated from the periodontal ligament

**Figure 1:**
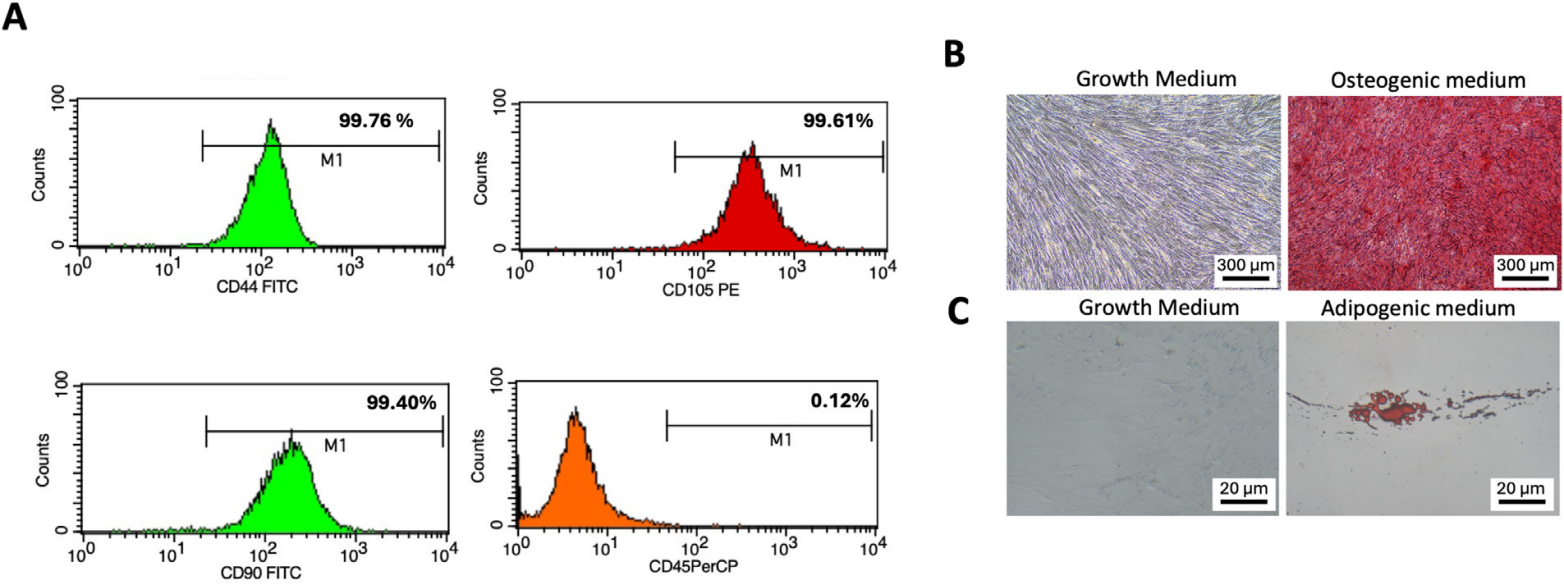
Periodontal ligament stem cells (PDLSCs) were characterised by analysing cell surface markers using flow cytometry (A). Mineralisation was assessed using Alizarin red staining after being cultured for 14 days (B). Intracellular lipid accumulation was observed using Oil Red O staining on day 16 (C).

The characteristics of periodontal ligament stem cells (PDLSCs) were investigated by analysing cell surface marker expression using flow cytometry. PDLSCs exhibited positive expression for mesenchymal stem cell surface markers, including CD44, CD73, and CD105, and CD45 was used as a negative control for the hematopoietic surface marker (Figure 1A). To investigate the multilineage differentiation potential of PDLSCs, osteogenic and adipogenic differentiation were performed using Alizarin Red S staining and Oil Red O staining, respectively. After culture in induction medium, PDLSCs exhibited increased mineralization compared with the control (Figure 1B). Similarly, intracellular lipid droplets were increased relative to the control (Figure 1C). These results indicated that the isolated PDLSCs represent a population of mesenchymal stem cells.

### Protein expression profiling of PTEN inhibition

**Figure 2:**
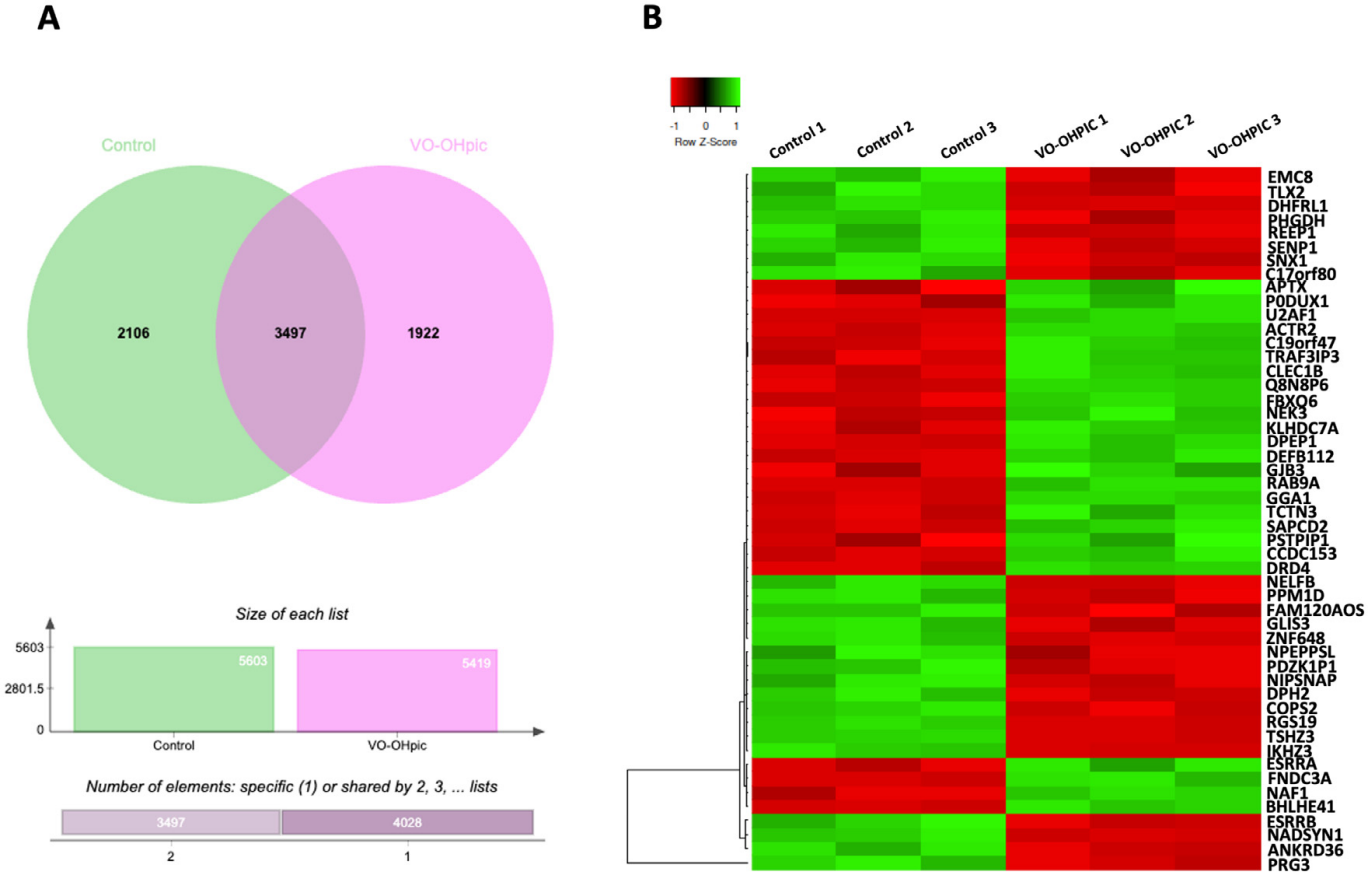
Protein profiling and pathway analysis of VO-OHpic-treated group (n = 3). Venn diagram of the protein in the untreated group and the VO-OHpic-treated group, based on raw protein counts (A). The top 50 differentially regulated proteins of the untreated group and the VO-OHpic-treated group (B). Gene lists were selected by *P*-values and analysed using Heatmapper. Row Z-scores in the heatmap show standardised expression for each gene across samples. Green indicates expression higher than that gene’s average, red indicates lower than average, and black indicates a value near zero.

Proteomic analysis was conducted to evaluate the effect of PTEN inhibition on protein expression in PDLSCs. Cells were treated with VO-OHpic for 24 hours in growth medium. The Venn diagram revealed that 2,106 proteins were uniquely expressed in the untreated group and 1,922 in the VO-OHpic-treated group, with 3,497 proteins commonly identified in both conditions (Figure 2A).

A total of 3,559 proteins were significant by FDR (Q < 0.05). Applying an additional fold-change cutoff (log2 FC > 1 or < -1), 2,306 proteins were identified as differentially expressed, including 1,435 upregulated and 871 downregulated proteins. The top 50 differentially regulated proteins were visualised in a Heatmap generated by the Heatmapper software,^16^ in which expression levels were represented as Z-scores to standardise raw protein abundance data into standard deviations (Figure 2B). Proteomic analysis revealed that proteins involved in cytoskeletal dynamics and vesicular transport (e.g., ACTR2, GGA1, RAB9A, FNDC3A, CCDC153), signal transduction and pathway modulation (e.g., TRAF3IP3, NEK3, ESRRA, DRD4), and cell communication and junction assembly (e.g., GJB3, CLEC1B, SAPCD2) were upregulated in the VO-OHpic–treated group. In contrast, several transcriptional regulators (e.g., TSHZ3, GLIS3, ZNF648, IKZF2, ESRRB) and proteins associated with membrane trafficking and endoplasmic reticulum function (e.g., SNX1, REEP1, EMC8) were downregulated following the VO-OHpic treatment.

### Enrichment analysis for upregulated and downregulated proteins

**Figure 3:**
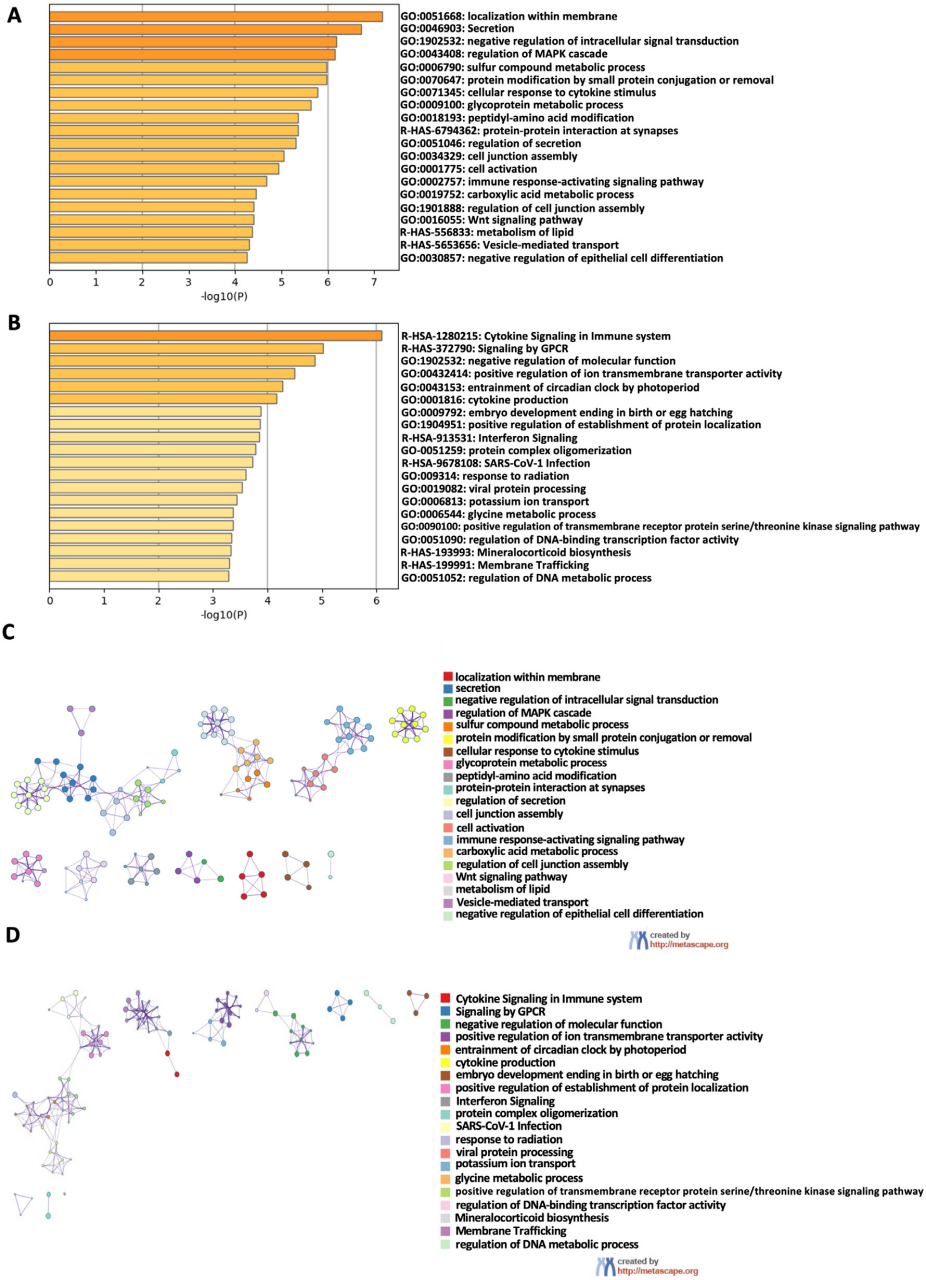
The Metascape analysis of differentially expressed proteins of the VO-OHpic-treated group (n = 3). demonstrated the bar graph of the top 20 enriched genes in the Pathway and Process Enrichment Analysis categories of differentially expressed proteins of the VO-OHpic-treated group compared to the untreated group, coloured by *P*-values upregulated (A) and downregulated (B). Protein-protein interaction (PPI) network of VO-OHpic-treated group, showing clustered enrichment categories and the relationship among these clusters (cluster ID) for upregulated proteins (C) and PPI network of downregulated proteins of VO-OHpic-treated group (D). All analyses were performed using Metascape.

Pathway and process enrichment analysis of differentially expressed proteins in the untreated group compared to the VO-OHpic-treated group was evaluated by Metascape of multiple gene lists,^22^ colored by *P*-values of <.05, with the following data sources from GO molecular function, GO biological processes, KEGG pathway, Reactome, and canonical pathway. The results were represented as clustered enrichment networks. The top 4 upregulated proteins in the VO-OHpic–treated group were associated with membrane localisation (GO:0051668), secretion (GO:0046903), negative regulation of intracellular signal transduction (GO:1902532), and regulation of the MAPK cascade (GO:0043408). Additionally, osteogenesis-related proteins involved in the Wnt signalling pathway (GO:0016055) were significantly upregulated in the treated group (Figure 3A). Conversely, the top 20 downregulated proteins identified through pathway and process enrichment analysis are shown in

Figure 3B, highlighting a marked downregulation of the cytokine signaling in the immune system pathway (R-HSA-1280125) in the VO-OHpic–treated group.

The protein-protein interaction networks in the VO-OHpic-treated group were identified and visualised as clustered enrichment categories, representing the relationship among functional groups. Highly expressed proteins associated with the regulation of secretion, vesicle-mediated transport, cell junction assembly, and synaptic protein interactions were grouped within the same cluster. Additionally, a distinct cluster was observed linking proteins involved in the regulation of the MAPK cascade and the negative regulation of intracellular signal transduction (Figure 3C). Among the significantly downregulated proteins, a cluster linking cytokine signaling in the immune system, membrane trafficking, and interferon signaling was observed in the VO-OHpic–treated group (Figure 3D).

**Figure 4:**
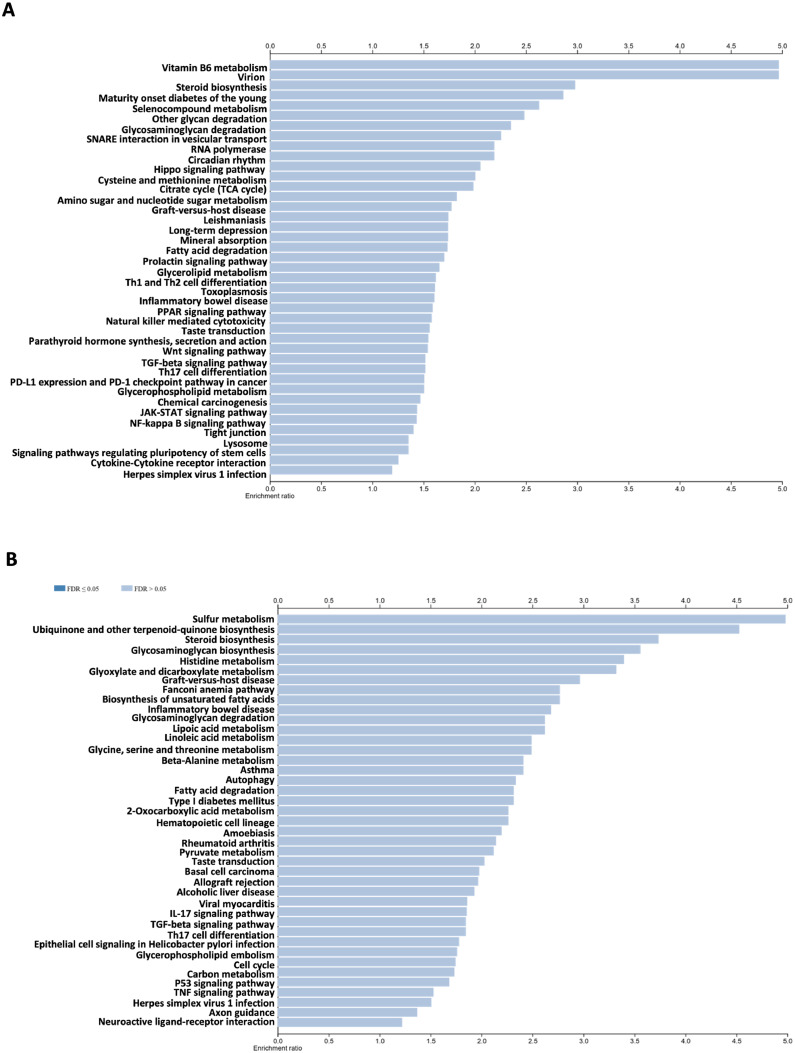
KEGG pathway analysis of VO-OHpic-treated group (n = 3) using Metascape gene list analysis. The KEGG-enriched pathways of VO-OHpic-treated group compared to the untreated group were analysed using WebGestalt. Upregulated protein expression in the KEGG-enriched pathways (A). Downregulated protein expression in the KEGG-enriched pathways (B). The analysis was conducted by assessing protein expression levels as log2 values, with significantly regulated proteins identified at an FDR-corrected *P*-value of < 0.05, representing pathway-associated protein expression.

Enriched KEGG pathways were evaluated using WebGestalt^23^ based on proteomic data expressed as log₂ fold-change values. Differentially expressed proteins in the VO-OHpic–treated group, compared with the untreated control, were mapped to corresponding KEGG pathways. Although trends toward both upregulation and downregulation of several pathways were observed in the VO-OHpic–treated group, none reached statistical significance (Figures 4A and 4B).

### Enrichment analysis for on-/off-expressed proteins

**Figure 5:**
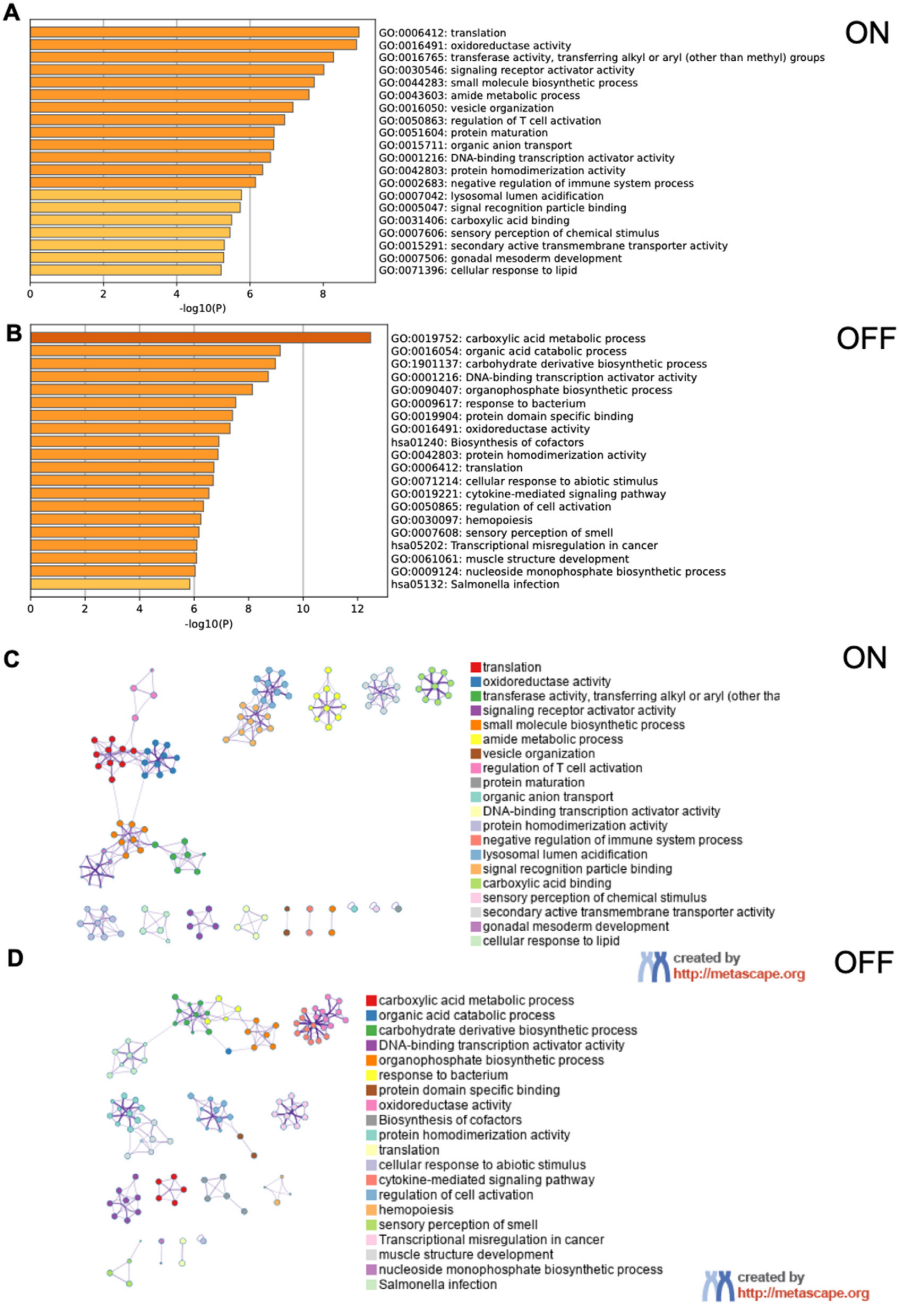
The Metascape analysis of on- and off-expressed proteins of the VO-OHpic-treated group (n = 3) demonstrated the bar graph of the top 20 enriched genes in the Pathway and Process Enrichment Analysis categories of differentially expressed proteins of the VO-OHpic-treated group compared to the untreated group, coloured by *P*-values on-target set (A) and off-target set (B). Protein-protein interaction (PPI) network of VO-OHpic-treated group, showing clustered enrichment categories and the relationship among these clusters (cluster ID) for on-target set proteins (C) and PPI network of off-target set proteins of VO-OHpic-treated group (D). All analyses were performed using Metascape.

**Figure 6:**
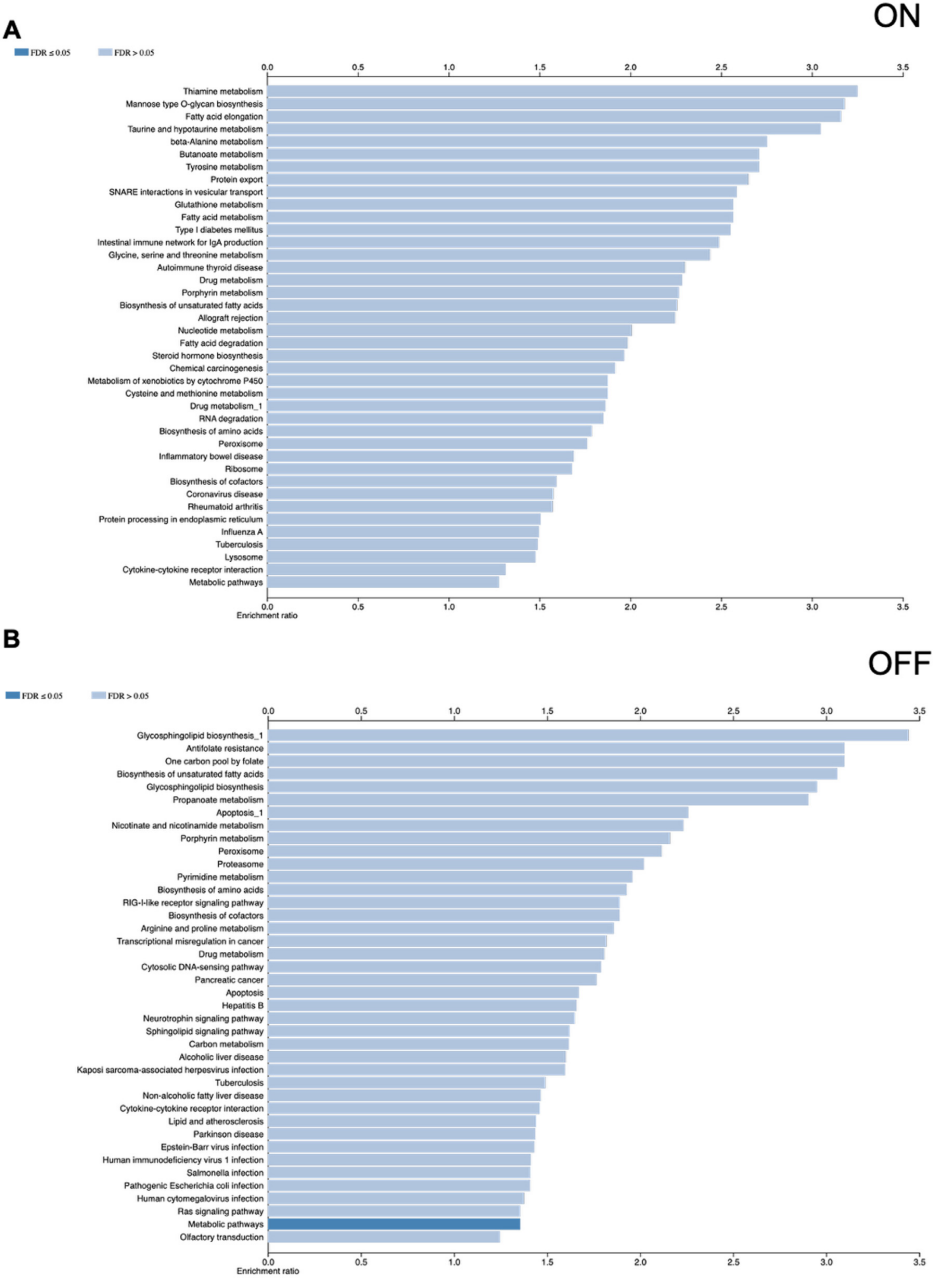
KEGG pathway analysis of VO-OHpic-treated group using Metascape analysis (n = 3). The KEGG-enriched pathways of VO-OHpic-treated group compared to the untreated group were analysed using WebGestalt. On-target set protein expression in the KEGG-enriched pathways (A). Off-target set protein expression in the KEGG-enriched pathways (B), representing pathway-associated protein expression.

To examine proteins detected in putative on- and off-target pathways after VO-OHpic exposure, the untreated and VO-OHpic-treated groups were compared in Metascape using the presence/absence of detectable proteins. In this analysis, 1,971 proteins were detected in the on-target set and 2,109 proteins in the off-target set. Within the on-target set, detected proteins mapped to terms including signalling receptor activator activity (GO:0030546) and regulation of T cell activation (GO:0050863) (Figure 5A). In the off-target set, detected proteins mapped to carboxylic acid metabolic process (GO:0019752) in the VO-OHpic group relative to control, suggesting potential alterations in metabolic processes following PTEN inhibition (Figure 5B). Protein–protein interaction networks for the on/off-target sets were visualised in Metascape as clustered enrichment categories and functional relationships (Figures 5C and 5D). KEGG pathway enrichment was assessed using WebGestalt.^23^ No KEGG pathways reached statistical significance for the on-target set (Figure 6A), whereas metabolic pathways were significantly enriched in the off-target set (Figure 6B).

### Effect of PTEN inhibition on gene expression and mineralisation in PDLSCs

**Figure 7:**
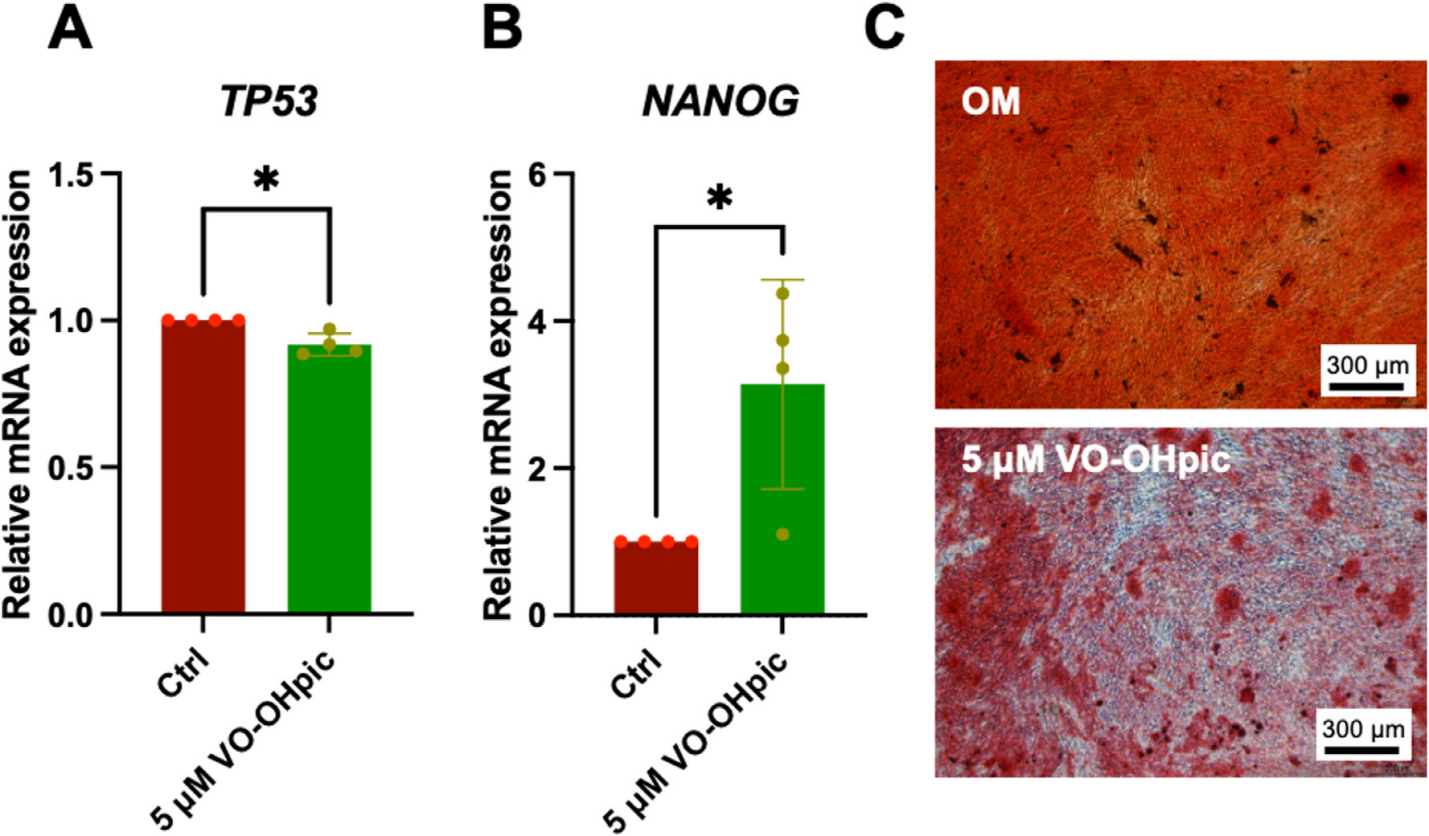
The functional assay of PTEN inhibitor, VO-OHpic, on PDLSCs. Cells were cultured in growth medium supplemented with 5 μM VO-OHpic. The mRNA expression of (A) *TP53* and (B) *NANOG* was examined by qRT-PCR at 24 h after treatment. For osteogenic differentiation, cells were cultured in osteogenic induction medium, with or without VO-OHpic (5 μM), for 14 days. The mineralisation was determined using Alizarin Red S staining (C). Each dot in (A) and (B) represents the raw data values from each donor (n = 4). The bars and asterisks indicated the statistically significant difference between groups (*P* < .05).

To investigate downstream targets of PTEN involved in cell proliferation and apoptosis in PDLSCs at the transcript level, cells were treated with the VO-OHpic (5μM) for 24 h in normal growth medium. The mRNA levels of *TP53* and *NANOG* were analysed by qRT-PCR. The results showed that *TP53* mRNA expression was significantly decreased in the treatment group compared with the control (Figure 7A). In contrast, *NANOG* mRNA expression was significantly upregulated compared with the control (Figure 7B). Further, cells were maintained in osteogenic induction in the presence or absence of VO-OHpic (5 μM) for 14 days. The mineral deposition was markedly attenuated in the VO-OHpic-treated group compared with the control (Figure 7C). Together, these results provide preliminary functional validation of PTEN inhibition in PDLSCs.

## Discussion

PTEN has been reported to play a key role in various biological processes in mesenchymal stem cells, including the regulation of inflammatory responses,^7^ cellular processes such as proliferation, differentiation, and cell survival,^6^ adipose tissue development and adipogenic differentiation,^24^ as well as osteogenesis differentiation.^5^ This study investigated proteomic alterations in periodontal ligament stem cells (PDLSCs) following PTEN inhibition with VO-OHpic to elucidate the molecular pathways underlying its biological effects. PTEN is a critical regulator of the PI3K/Akt and mitogen-activated protein kinases (MAPK) signalling pathways, and its inhibition is known to enhance cell proliferation, survival, and differentiation.^25^^,^^26^ Our proteomic findings reveal that 2,306 proteins were differentially expressed, including 1,435 upregulated and 871 downregulated proteins. 1,971 proteins were detected in the on-target set and 2,109 proteins in the off-target set. This evidence indicates directionally biased proteomic changes, providing support for the notion that PTEN inhibition triggers coordinated proteomic transitions, thereby supporting its role as a modulator of PDLSC behaviours.

The differentially expressed proteins revealed distinct biological shifts between treated and untreated groups. Proteins related to cytoskeletal dynamics, vesicular transport, signal transduction, and cell–cell communication (e.g., ACTR2, GGA1, RAB9A, FNDC3A, TRAF3IP3, and GJB3) were markedly upregulated in VO-OHpic–treated PDLSCs. ACTR2 (actin-related protein 2) is a core component of the Arp2/3 complex, which drives actin nucleation and filament branching; essential for cytoskeletal remodelling, cell migration, and adhesion.^27^ FNDC3A (fibronectin type-III domain-containing protein 3A) contributes to cell morphology and adhesion by mediating actin–membrane interactions and has been implicated in cellular differentiation processes.^28^^,^^29^ GGA1 (Golgi-associated gamma-adaptin ear-containing ARF-binding protein 1) and RAB9A (member of the Rab GTPase family) regulate vesicle-mediated trafficking between the Golgi apparatus and endosomes, facilitating protein sorting and receptor recycling.^30^^,^^31^ These vesicular and cytoskeletal processes are crucial for maintaining receptor localisation and turnover, which in turn facilitate MAPK signalling activation.^32^^,^^33^ Thus, PTEN inhibition appears to enhance membrane trafficking and signalling readiness in PDLSCs.

This study used Metascape to provide a comprehensive overview of enriched biological pathways by integrating multiple gene or protein lists, enabling intuitive visualisation and interpretation. Metascape-generated bar graphs display the top non-redundant enrichment clusters, each represented by a single term, including protein–protein interactions.^19^ This approach helps elucidate potential functional roles, protein interactions, and signalling cascades associated with PTEN signalling. However, the pathways identified as significantly enriched by Metascape reflect statistical associations among genes/proteins, and their biological relevance should be interpreted cautiously and validated through functional studies. In the present study, PTEN inhibition may contribute to stemness, cell proliferation, and osteogenic differentiation, as it decreased *TP53* mRNA expression, increased *NANOG* mRNA expression, and attenuated mineral deposition in PDLSCs. Thus, although the enriched pathway showed no significant difference, it still provided a clue for further biological and functional investigation.

Pathway enrichment analysis further supported the upregulation of the MAPK cascade (GO:0043408) and the negative regulation of intracellular signal transduction, reflecting coordinated activation and fine-tuning of signalling dynamics. As a central intracellular signalling mechanism, MAPK pathways integrate multiple cellular responses, including proliferation, differentiation, stress adaptation, and anti-inflammatory regulation and are interconnected with ERK1/2, p38 MAPK, and JNK cascades.^34^, ^35^, ^36^, ^37^ Previous studies have reported that the loss of PTEN in breast cancer progression promotes a crosstalk network between the PI3K/Akt and MAPK signalling pathways, thereby regulating tumour survival and proliferation.^38^ The MAPK signalling cascade in the intracellular signalling system also plays an essential role in odontoblast differentiation through the ERK pathway, which is involved in late-stage osteogenic differentiation.^39^ Interestingly, inhibition of ERK signalling has been shown to suppress the osteogenic differentiation while promoting the later stages of osteoblast differentiation. Moreover, the ERK/MAPK signalling pathway can be activated by substrates such as Mg, Ca, and Sr ions, which bind to cell-surface receptors, thereby promoting osteoblast proliferation and differentiation via Wnt/β-Catenin.^40^ Furthermore, the ERK signalling pathway and the MAPK cascade are directly involved in regulating cell proliferation and differentiation through growth factor stimulation. These pathways function through a phosphorylation cascade that sequentially activates downstream kinases.^41^ The ERK/MAPK signalling pathway mediates osteogenic differentiation by significantly enhancing *RUNX2* and *BSP* expression through TNF-α- mediated positive regulation in osteoblast precursor cells.^42^ In our previous findings, VO-OHpic treatment in PDLSCs rescued ERK signalling, as determined by transcriptomic analysis (GSE295018), further suggesting that PTEN inhibition may be involved in osteogenic differentiation through modulation of the ERK/MAPK pathway.

Additionally, the Wnt signalling pathway (GO:0016055) was significantly upregulated in VO-OHpic-treated PDLSCs. The Wnt/β-catenin pathway is a critical regulator of stem cell self-renewal and periodontal tissue homeostasis.^43^, ^44^, ^45^ PTEN has been reported to act as a negative regulator of the Wnt/β-catenin signalling pathway by inhibiting β-catenin and Cyclin D1, particularly in cancer and developmental contexts.^46^^,^^47^ Therefore, PTEN inhibition may relieve this suppression, enhance Wnt/β-catenin signalling, and possibly promote the osteogenic potential of PDLSCs. However, further investigation is required to clarify the detailed mechanisms underlying this observation.

On the downregulation side, several transcriptional regulators (e.g., TSHZ3, GLIS3, ZNF648, IKZF2, and ESRRB) and proteins involved in membrane trafficking and endoplasmic reticulum function (e.g., SNX1, REEP1, and EMC8) were significantly downregulated following VO-OHpic treatment. These changes reflect reduced immune and stress-related signalling, as SNX1 and REEP1 are known to mediate cytokine receptor recycling and membrane protein localisation.^48^, ^49^, ^50^ The decline in GLIS3, a zinc finger transcription factor associated with developmental and metabolic control,^51^ together with reduced expression of PHGDH and NADSYN1, enzymes central to serine biosynthesis^52^^,^^53^ and NAD⁺ metabolism,^54^^,^^55^ suggests a metabolic reprogramming toward decreased anabolic flux.

Additionally, the downregulation of ESRRB, an orphan nuclear receptor critical for pluripotency maintenance,^56^ indicate a loss of stemness and potential commitment to lineage-specific differentiation. ESRRB is a member of the nuclear receptor family, known to regulate energy metabolism,^57^^,^^58^ self-renewal in embryo-derived stem cells,^59^ and enhance cellular reprogramming.^57^^,^^60^ This shift aligns with the behaviour of mesenchymal stem cells, which transition from proliferation and stress responses toward a differentiation-primed phenotype.^61^ Notably, ESRRB function is modulated by ERK-mediated phosphorylation, which balances self-renewal and differentiation in mouse embryonic stem cells.^62^ Thus, the reduced ESRRB expression in the VO-OHpic group may indicate altered ERK-dependent signalling and may be linked to differentiation regulation. However, these effects can vary across cell types, and the specific role of ESRRB in PDLSCs remains unclear and indeed requires further investigation to confirm these hypotheses.

PTEN itself plays a crucial role in directly inhibiting the inflammatory response and regulating inflammatory cytokines through T- and B-cell receptors, cytokine receptor molecules, integrins, and also growth factor receptors, specifically M1 and M2 inflammatory cytokines.^7^^,^^63^ A previous study reported that PTEN is important for the inhibition of inflammation and alveolar bone loss in periodontitis, which is suppressed by inhibiting interleukin 1 (IL-1) and tumour necrosis factor (TNF-α) pathways.^9^ Consistent with these findings, pathway enrichment analysis in the present study revealed that the top 20 downregulated proteins were linked to cytokine signalling in the immune system (R-HSA-1280125) and cytokine production (GO:0001816). Downregulation of these immune-associated pathways, together with decreased interferon signalling, suggests a quiescent, anti-inflammatory state with diminished receptor turnover.

Although KEGG pathway enrichment revealed no statistically significant changes in our study, the trends observed toward both upregulated and downregulated networks suggest selective proteomic adaptation rather than global pathway remodelling. This fine-tuned response reflects the dual function of PTEN in maintaining cellular homeostasis, balancing survival and differentiation signals while restraining excessive immune activation in PDLSCs.

PTEN is a central regulator of cellular metabolism and energy homeostasis.^2^^,^^64^ Our results show that metabolic pathways were enriched among the predicted off-target pathways, suggesting that PTEN inhibition is associated with altered regulation of metabolic programs. Consistent with this, studies using PTEN-deficient mouse models have reported that constitutive PTEN loss can disrupt mitochondrial function, including effects on the tricarboxylic acid (TCA) cycle and oxidative phosphorylation, and promote a shift toward glycolytic metabolism. These changes have been associated with increased oxidative stress, elevated reactive oxygen species (ROS), and genomic damage, which may contribute to pathological outcomes such as tumorigenesis.^65^^,^^66^ Notably, the metabolic pathway signals identified here included altered expression of several sirtuin family members (SIRT1, SIRT3, SIRT6, and SIRT7). Sirtuins are NAD^+^-dependent deacylases that regulate metabolic adaptation in a compartment-specific manner (e.g., SIRT1 predominantly nuclear/cytosolic; SIRT3 mitochondrial). SIRT1 has been reported to deacetylate β-catenin and modulate its transcriptional activity, thereby influencing mesenchymal stem cell (MSC) differentiation.^67^ In addition, SIRT1 can regulate PTEN through deacetylation, affecting PTEN activity and subcellular localisation in contexts linked to cellular redox status and ROS signalling. Together, these results support a model in which PTEN inhibition perturbs metabolic signalling networks and mitochondrial energy metabolism, potentially compromising cellular metabolic control and homeostasis.

Periodontal ligament stem cells (PDLSCs) play an essential role in periodontal tissue regeneration, which aims to restore damaged or diseased periodontal ligament (PDL) tissues to their original structure and function.^9^ This regenerative process involves the coordinated repair of both hard and soft tissues, including the periodontal ligament, cementum, and alveolar bone.^68^^,^^69^ Previous studies have reported that decellularized extracellular matrix (dECM) derived from inflammation-conditioned PDL tissues supports cell growth and differentiation and may serve as an alternative biomaterial for regenerative therapies.^14^ In this context, PTEN inhibition may alter extracellular matrix–related proteins, thereby influencing periodontal tissue remodelling and regenerative capacity. In addition to their regenerative potential, PDLSCs exhibit immunomodulatory functions by regulating the activity of various immune cells, including macrophages, granulocytes, dendritic cells, B cells, and T cells.^70^ The upregulation of immunoregulatory factors such as indoleamine 2,3-dioxygenase (IDO), transforming growth factor–β1 (TGF-β1), and hepatocyte growth factor (HGF) has been shown to contribute to the immunosuppressive properties of mesenchymal stem cells, including PDLSCs.^71^ In the present study, proteomic analysis revealed that PTEN inhibition in PDLSCs was associated with changes in proteins related to osteogenesis, mineralisation, and immunomodulation, as well as signalling pathways including PI3K/Akt, MAPK, Wnt, and TGF-β. These biological processes are closely linked to periodontal tissue regeneration. In the present study, we demonstrated that PTEN inhibition increased *NANOG* expression and was associated with reduced mineral deposition in PDLSCs, suggesting a role in regulating stemness and osteogenic differentiation. However, our osteogenic induction experiments used continuous treatment; future studies should also evaluate pre-treatment (priming) protocols, as timing can influence osteogenic outcomes (e.g., TGF-β-mediated modulation). In addition, the contribution of PTEN to immunomodulation warrants further investigation, including the role of PTEN inhibition on cytokine expression and its indirect influence on immune cell functions.

The present study also preliminary investigated the functions of VO-OHpic in PDLSCs. qRT-PCR validation of selected targets of PTEN downstream at the transcript level was evaluated to assess and preliminarily investigate the effect of PTEN on cell proliferation and apoptosis in PDLSC. p53 is a downstream target of PTEN, and PTEN modulates p53 protein levels and transcriptional activity through both phosphatase-dependent and -independent mechanisms, thereby regulating cell growth, survival, and death.^72^ *NANOG* is a key transcription factor involved in the self-renewal of embryonic stem cells (ESCs) and in maintaining stem cell characteristics.^73^ In a previous study, PTEN deficiency was reported to alter Nanog expression, thereby significantly upregulating self-renewal and promoting stem cell properties.^74^^,^^75^ Consistently, this study showed that *TP53* mRNA expression was significantly reduced, whereas NANOG expression was significantly increased, in the treatment group compared with the control group. The impact of PTEN inhibition on osteogenic differentiation was also assessed. In this regard, 5 μM VO-OHpic markedly decreased mineralised nodule formation in PDLSCs, consistent with a previous study reporting that PTEN inhibition reduces the osteogenic differentiation capacity of human dental pulp cells.^10^ Collectively, these results indicate that VO-OHpic influences PDLSC proliferation and osteogenic differentiation.

Although VO-OHpic is widely used as a PTEN inhibitor, it can also inhibit other phosphatases, including tyrosine phosphatase SHP1 and the phosphoinositide phosphatases INPP4A and INPP4B.^76^ Similar off-target effects have been reported for other commonly used PTEN inhibitors, such as bpV(phen), bpV(pic), and SF1670.^76^ Nevertheless, VO-OHpic appears more specific for PTEN than bpVpic, with IC50 values for other enzymes ∼13–20-fold higher.^77^ Therefore, VO-OHpic may be the preferable choice among available compounds, but because off-target activity cannot be fully ruled out, results should be interpreted with caution.

The present study has several limitations. First, it used cells isolated from only 3 donors. This relatively small sample size may reduce statistical power to detect differences in protein expression between groups, thereby limiting the generalizability of the findings. Nonetheless, the study offers important biological insights into PTEN's role in PDLSCs. Future functional studies with larger sample sizes would help confirm these biological effects. Second, this study tested only a single VO-OHpic concentration. In our earlier work, human dental pulp cells and human adipose tissue-derived stromal cells were exposed to multiple VO-OHpic doses (0.625, 1.25, 2.5, and 5 μM), none of which were toxic at 24 h. However, 5 μM VO-OHpic reduced proliferation in human dental pulp cells by day 7, reflected by decreased colony formation and a smaller S-phase population. Further, it also markedly reduced intracellular lipid accumulation in human adipose tissue-derived stromal cells.^10^ On this basis, 5 μM was chosen as the optimal dose for PTEN-associated proteomic analysis in the present study. Finally, only one time point was examined to assess proteomic changes, potentially limiting the evaluation of dynamic, time-dependent protein alterations following PTEN inhibition. Future studies should therefore investigate the effects of PTEN inhibition in PDLSCs across multiple time points.

In conclusion, PTEN inhibition by VO-OHpic was associated with a coordinated proteomic response in PDLSCs, implicated by the activation of cytoskeletal remodelling, vesicle-mediated trafficking, and MAPK/Wnt signalling, alongside the suppression of transcriptional, metabolic, and immune-regulatory networks. These patterns suggest that PTEN may contribute to modulating inflammatory signalling and regenerative functions in PDLSCs, despite the fact that causality cannot be inferred from proteomics alone. The concurrent regulation of MAPK/Wnt-related pathways and cytokine signalling pathways might imply a transition from an immune-reactive to a pro-regenerative phenotype. Overall, these proteomic changes may influence differentiation and tissue repair, providing further clues into PTEN-dependent signalling mechanisms that govern stem cell behaviour and periodontal regeneration. Further studies are warranted to delineate the downstream molecular interactions and to validate these findings *in vivo*.

## Author contributions

**Suphalak Phothichailert:** Collected literature, contributed to methodology, data acquisition, analysis, interpretation, drafted and critically revised the manuscript. **Nunthawan Nowwarote:** Contributed to methodology, data analysis and critically revised manuscript. **Chatvadee Kornsuthisopon:** Contributed to data acquisition and critically revised manuscript. **Shinya Murakami:** Contributed to manuscript editing and critically revised manuscript. **Supreda Suphanantachat Srithanyarat:** Contributed to conceptualization, data analysis and interpretation, drafted and critically revised the manuscript. **Thanaphum Osathanon:** Contributed to conception and design, data analysis and interpretation and critically revised the manuscript.

## Ethics approvals

The protocol is approved by the Human Research Ethics Research Committee, Faculty of Dentistry, Chulalongkorn University (approval No. HREC-DCU 2025-006).

## Availability of data and materials

The datasets generated during the current study are available in the ProteomeXchange via the PRIDE database repository under the accession number PXD074161.

## Funding

The present study was supported by the Faculty of Dentistry Research Fund, Chulalongkorn University to T.O. (DRF 69_003). S.P. was supported by the Second Century Fund, Chulalongkorn University (C2F PhD Scholarship).

## Declaration of generative AI and AI-assisted technologies in the writing process

During the revision of this manuscript, the authors used ChatGPT-5.2 to improve the clarity and readability of selected sections of the text. The tool was applied solely for language editing, not for the generation of scientific content. All changes were carefully reviewed and finalised by the authors, who take full responsibility for the accuracy and integrity of the manuscript.

## Declaration of competing interest

The authors declare the following financial interests/personal relationships which may be considered as potential competing interests: Prof. Thanaphum Osathanon (co-author) is an editorial board member of International Dental Journal. Given his role as co-author, he had no involvement in the peer review of this article and had no access to information regarding its peer review. Full responsibility for the editorial process for this article was delegated to another journal editor. If there are other authors, they declare that they have no known competing financial interests or personal relationships that could have appeared to influence the work reported in this paper.

## Appendix Supplementary materials

Image, application 1

## Footnotes

Supplementary material associated with this article can be found in the online version at doi:10.1016/j.identj.2026.109493.

## References

[1] Pulido R., Baker S.J., Barata J.T., Carracedo A., Cid V.J., Chin-Sang I.D. (2014). A unified nomenclature and amino acid numbering for human pten. Science Signaling.

[2] Chen C.Y., Chen J., He L., Stiles BL. (2018). PTEN: tumor suppressor and metabolic regulator. Front Endocrinol (Lausanne).

[3] Sun Y., Tian H., Wang L. (2015). Effects of PTEN on the proliferation and apoptosis of colorectal cancer cells via the phosphoinositol-3-kinase/Akt pathway. Oncol Rep.

[4] Downes C.P., Ross S., Maccario H., Perera N., Davidson L., Leslie NR. (2007). Stimulation of PI 3-kinase signaling via inhibition of the tumor suppressor phosphatase, PTEN. Adv Enzyme Regul.

[5] Liu X., Chen T., Wu Y., Tang Z. (2017). Role and mechanism of PTEN in adiponectin-induced osteogenesis in human bone marrow mesenchymal stem cells. Biochem Biophys Res Commun.

[6] Kero D., Cigic L., Medvedec Mikic I., Galic T., Cubela M., Vukojevic K. (2016). Involvement of IGF-2, IGF-1R, IGF-2R and PTEN in development of human tooth germ - an immunohistochemical study. Organogenesis.

[7] Johnson T.A., Singla DK. (2018). PTEN inhibitor VO-OHpic attenuates inflammatory M1 macrophages and cardiac remodeling in doxorubicin-induced cardiomyopathy. Am J Physiol Heart Circ Physiol.

[8] Liu X., Bruxvoort K.J., Zylstra C.R., Liu J., Cichowski R., Faugere M.C. (2007). Lifelong accumulation of bone in mice lacking Pten in osteoblasts. Proc Natl Acad Sci U S A..

[9] Fu C., Wei Z., Zhang D. (2019). PTEN inhibits inflammatory bone loss in ligature-induced periodontitis via IL1 and TNF-alpha. Biomed Res Int.

[10] Nowwarote N., Osathanon T., Fournier B.P.J., Theerapanon T., Yodsanga S., Kamolratanakul P. (2023). PTEN regulates proliferation and osteogenesis of dental pulp cells and adipogenesis of human adipose-derived stem cells. Oral Dis.

[11] Al-Amrani S., Al-Jabri Z., Al-Zaabi A., Alshekaili J., Al-Khabori M. (2021). Proteomics: concepts and applications in human medicine. World J Biol Chem.

[12] Chakraborty S., Hosen M.I., Ahmed M., Shekhar HU. (2018). Onco-multi-OMICS approach: a new frontier in cancer research. Biomed Res Int.

[13] Zhang J., Kim S., Li L., Kemp C.J., Jiang C., Lü J. (2020). Proteomic and transcriptomic profiling of Pten gene-knockout mouse model of prostate cancer. Prostate.

[14] Phothichailert S., Kornsuthisopon C., Chansaenroj A., Trachoo V., Nowwarote N., Fournier B. (2025). Decellularised matrices from force loaded periodontal ligament stem cells support osteogenic differentiation. Scientific Reports.

[15] Liu S., Dang L., Guo X., Wu K., Qu X., Xu J. (2025). Targeting MSR1 to facilitate efferocytosis: a novel strategy for immune homeostasis regulation in irreversible pulpitis. Int Dent J.

[16] Babicki S., Arndt D., Marcu A., Liang Y., Grant J.R., Maciejewski A. (2016). Heatmapper: web-enabled heat mapping for all. Nucleic Acids Res.

[17] Bardou P., Mariette J., Escudié F., Djemiel C., Klopp C. (2014). jvenn: an interactive Venn diagram viewer. BMC Bioinformatics.

[18] Liao Y., Wang J., Jaehnig E.J., Shi Z., Zhang B. (2019). WebGestalt 2019: gene set analysis toolkit with revamped UIs and APIs. Nucleic Acids Res.

[19] Zhou Y., Zhou B., Pache L., Chang M., Khodabakhshi A.H., Tanaseichuk O. (2019). Metascape provides a biologist-oriented resource for the analysis of systems-level datasets. Nat Commun.

[20] Shannon P., Markiel A., Ozier O., Baliga N.S., Wang J.T., Ramage D. (2003). Cytoscape: a software environment for integrated models of biomolecular interaction networks. Genome Res.

[21] Chen L., Liu L., Lin T., Mai Z., Lu H., Hu B. (2025). HDAC9-mediated pyroptosis promotes orthodontically induced inflammatory root resorption. Int Dent J.

[22] Zhou Y., Zhou B., Pache L., Chang M., Khodabakhshi A.H., Tanaseichuk O. (2019). Metascape provides a biologist-oriented resource for the analysis of systems-level datasets. Nature Commun.

[23] Elizarraras J.M., Liao Y., Shi Z., Zhu Q., Pico A.R., Zhang B. (2024). WebGestalt 2024: faster gene set analysis and new support for metabolomics and multi-omics. Nucleic Acids Res.

[24] Huang W., Queen N.J., McMurphy T.B., Ali S., Cao L. (2019). Adipose PTEN regulates adult adipose tissue homeostasis and redistribution via a PTEN-leptin-sympathetic loop. Mol Metab.

[25] Salmena L., Carracedo A., Pandolfi PP. (2008). Tenets of PTEN tumor suppression. Cell.

[26] Song M.S., Salmena L., Pandolfi PP. (2012). The functions and regulation of the PTEN tumour suppressor. Nat Rev Mol Cell Biol.

[27] Pollard T.D., Borisy GG. (2003). Cellular motility driven by assembly and disassembly of actin filaments. Cell.

[28] Jiang H., Chu B.L., He J., Liu Z., Yang L. (2022). Expression and prognosis analyses of the fibronectin type-III domain-containing (FNDC) protein family in human cancers: A Review. Medicine (Baltimore).

[29] Obholz K.L., Akopyan A., Waymire K.G., MacGregor GR. (2006). FNDC3A is required for adhesion between spermatids and Sertoli cells. Dev Biol.

[30] Bonifacino JS. (2004). The GGA proteins: adaptors on the move. Nat Rev Mol Cell Biol.

[31] Stenmark H. (2009). Rab GTPases as coordinators of vesicle traffic. Nat Rev Mol Cell Biol.

[32] Clevers H., Nusse R. (2012). Wnt/β-catenin signaling and disease. Cell.

[33] Sorkin A., von Zastrow M. (2009). Endocytosis and signalling: intertwining molecular networks. Nat Rev Mol Cell Biol.

[34] Keshet Y., Seger R. (2010). The MAP kinase signaling cascades: a system of hundreds of components regulates a diverse array of physiological functions. Methods Mol Biol.

[35] Atkins C.M., Selcher J.C., Petraitis J.J., Trzaskos J.M., Sweatt JD. (1998). The MAPK cascade is required for mammalian associative learning. Nat Neurosci.

[36] Zhan X., Li J., Zhou T. (2021). Targeting Nrf2-mediated oxidative stress response signaling pathways as new therapeutic strategy for pituitary adenomas. Front Pharmacol.

[37] Kim HK. (2014). Role of ERK/MAPK signalling pathway in anti-inflammatory effects of Ecklonia cava in activated human mast cell line-1 cells. Asian Pacific J Trop Med.

[38] Ebbesen S.H., Scaltriti M., Bialucha C.U., Morse N., Kastenhuber E.R., Wen H.Y. (2016). Pten loss promotes MAPK pathway dependency in HER2/neu breast carcinomas. Proc Natl Acad Sci U S A..

[39] Murakami S., Noguchi T., Takeda K., Ichijo H. (2007). Stress signaling in cancer. Cancer Sci.

[40] Nie X., Zhang X., Lei B., Shi Y., Yang J. (2022). Regulation of magnesium matrix composites materials on bone immune microenvironment and osteogenic mechanism. Front Bioeng Biotechnol.

[41] Haq R., Brenton J.D., Takahashi M., Finan D., Finkielsztein A., Damaraju S. (2002). Constitutive p38HOG mitogen-activated protein kinase activation induces permanent cell cycle arrest and senescence. Cancer Res.

[42] Zhang Y., Yang C., Ge S., Wang L., Zhang J., Yang P. (2020). EphB4/TNFR2/ERK/MAPK signaling pathway comprises a signaling axis to mediate the positive effect of TNF-α on osteogenic differentiation. BMC Mol Cell Biol.

[43] Yu M., Qin K., Fan J., Zhao G., Zhao P., Zeng W. (2024). The evolving roles of Wnt signaling in stem cell proliferation and differentiation, the development of human diseases, and therapeutic opportunities. Genes Dis.

[44] Nusse R. (2008). Wnt signaling and stem cell control. Cell Res.

[45] Bao J., Yang Y., Xia M., Sun W., Chen L. (2021). Wnt signaling: An attractive target for periodontitis treatment. Biomed Pharmacother.

[46] Gong J., Shen Y., Jiang F., Wang Y., Chu L., Sun J. (2022). MicroRNA-20a promotes non-small cell lung cancer proliferation by upregulating PD-L1 by targeting PTEN. Oncol Lett.

[47] Persad A., Venkateswaran G., Hao L., Garcia M.E., Yoon J., Sidhu J. (2016). Active beta-catenin is regulated by the PTEN/PI3 kinase pathway: a role for protein phosphatase PP2A. Genes Cancer.

[48] Cullen PJ. (2008). Endosomal sorting and signalling: an emerging role for sorting nexins. Nat Rev Mol Cell Biol.

[49] Cullen P.J., Steinberg F. (2018). To degrade or not to degrade: mechanisms and significance of endocytic recycling. Nat Rev Mol Cell Biol.

[50] Björk S., Hurt C.M., Ho V.K., Angelotti T. (2013). REEPs are membrane shaping adapter proteins that modulate specific g protein-coupled receptor trafficking by affecting ER cargo capacity. PLoS One.

[51] Kim Y.S., Nakanishi G., Lewandoski M., Jetten AM. (2003). GLIS3, a novel member of the GLIS subfamily of Krüppel-like zinc finger proteins with repressor and activation functions. Nucleic Acids Res.

[52] Mattaini K.R., Sullivan M.R., Vander Heiden M.G. (2016). The importance of serine metabolism in cancer. J Cell Biol.

[53] Reid M.A., Allen A.E., Liu S., Liberti M.V., Liu P., Liu X. (2018). Serine synthesis through PHGDH coordinates nucleotide levels by maintaining central carbon metabolism. Nature Commun.

[54] Nikiforov A., Kulikova V., Ziegler M. (2015). The human NAD metabolome: functions, metabolism and compartmentalization. Crit Rev Biochem Mol Biol.

[55] Zapata-Pérez R., Wanders R.J.A., van Karnebeek C.D.M., Houtkooper RH. (2021). NAD(+) homeostasis in human health and disease. EMBO Mol Med.

[56] Festuccia N., Osorno R., Halbritter F., Karwacki-Neisius V., Navarro P., Colby D. (2012). Esrrb is a direct Nanog target gene that can substitute for Nanog function in pluripotent cells. Cell Stem Cell.

[57] Festuccia N., Owens N., Navarro P. (2018). Esrrb, an estrogen-related receptor involved in early development, pluripotency, and reprogramming. FEBS Letters.

[58] Yu S., Zhang R., Shen Q., Zhu Z., Zhang J., Wu X. (2021). ESRRB facilitates the conversion of trophoblast-like stem cells from induced pluripotent stem cells by directly regulating CDX2. Front Cell Dev Biol.

[59] Okamura E., Tam O.H., Posfai E., Li L., Cockburn K., Lee C.Q.E. (2019). Esrrb function is required for proper primordial germ cell development in presomite stage mouse embryos. Develop Biol.

[60] Buganim Y., Faddah Dina A., Cheng Albert W., Itskovich E., Markoulaki S., Ganz K. (2012). Single-cell expression analyses during cellular reprogramming reveal an early stochastic and a late hierarchic phase. Cell.

[61] Zhang J., Zhao J., Dahan P., Lu V., Zhang C., Li H. (2018). Metabolism in pluripotent stem cells and early mammalian development. Cell Metab.

[62] Duan X., Zhang Q., Gao L., Ling B., Du X., Chen L. (2025). ERK phosphorylates ESRRB to regulate the self-renewal and differentiation of mouse embryonic stem cells. Stem Cell Reports.

[63] Taylor H., Laurence A.D.J., Uhlig HH. (2019). The Role of PTEN in Innate and Adaptive Immunity. Cold Spring Harb Perspect Med.

[64] Aquila S., Santoro M., Caputo A., Panno M.L., Pezzi V., De Amicis F. (2020). The tumor suppressor PTEN as molecular switch node regulating cell metabolism and autophagy: implications in immune system and tumor microenvironment. Cells.

[65] Antico Arciuch V.G., Russo M.A., Kang K.S., Di Cristofano A. (2013). Inhibition of AMPK and Krebs cycle gene expression drives metabolic remodeling of Pten-deficient preneoplastic thyroid cells. Cancer Res.

[66] Bankoglu E.E., Tschopp O., Schmitt J., Burkard P., Jahn D., Geier A. (2016). Role of PTEN in oxidative stress and DNA damage in the liver of whole-body Pten haplodeficient mice. PLoS One.

[67] Nogueiras R., Habegger K.M., Chaudhary N., Finan B., Banks A.S., Dietrich M.O. (2012). Sirtuin 1 and sirtuin 3: physiological modulators of metabolism. Physiol Rev.

[68] Xiong J., Menicanin D., Zilm P.S., Marino V., Bartold P.M., Gronthos S. (2016). Investigation of the cell surface proteome of human periodontal ligament stem cells. Stem Cells Int.

[69] Han P., Ivanovski S., Crawford R., Xiao Y. (2015). Activation of the canonical Wnt signaling pathway induces cementum regeneration. J Bone Miner Res.

[70] Andrukhov O., Behm C., Blufstein A., Rausch-Fan X. (2019). Immunomodulatory properties of dental tissue-derived mesenchymal stem cells: implication in disease and tissue regeneration. World J Stem Cells.

[71] Wada N., Menicanin D., Shi S., Bartold P.M., Gronthos S. (2009). Immunomodulatory properties of human periodontal ligament stem cells. J Cell Physiol.

[72] Freeman D.J., Li A.G., Wei G., Li H-H, Kertesz N., Lesche R. (2003). PTEN tumor suppressor regulates p53 protein levels and activity through phosphatase-dependent and -independent mechanisms. Cancer Cell.

[73] Li Y., Ma R., Hao X. (2024). Therapeutic role of PTEN in tissue regeneration for management of neurological disorders: stem cell behaviors to an in-depth review. Cell Death Dis.

[74] Pulido R. (2018). PTEN inhibition in human disease therapy. Molecules.

[75] Zhang G., Wang W., Yao C., Zhang S., Liang L., Han M. (2017). Radiation-resistant cancer stem-like cell properties are regulated by PTEN through the activity of nuclear β-catenin in nasopharyngeal carcinoma. Oncotarget.

[76] Spinelli L., Lindsay Y.E., Leslie NR. (2015). PTEN inhibitors: An evaluation of current compounds. Adv Biol Regul.

[77] Mak L.H., Woscholski R. (2015). Targeting PTEN using small molecule inhibitors. Methods.

