# Supplementary material for: Proteomic Profiling of PTEN Inhibition on Periodontal Ligament Stem Cells
Source: Int Dent J. 2026 Mar 20;76(3):109493. doi: 10.1016/j.identj.2026.109493 (PMC13019790; doi:10.1016/j.identj.2026.109493)
Supplement: Supplementary file 1 [file mmc1.docx]

Supplementary file 1

KEGG pathway by WebGestalt gene lists (Upregulate)

ID: hsa04392; Name: Hippo signaling pathway

| Gene Symbol | Gene name | log2FoldChange | p-value |
| --- | --- | --- | --- |
| AJUBA | ajuba LIM protein | 1.2011 | 6.55E-01 |
| YAP1 | Yes1 associated transcriptional regulator | 2.4875 | 1.59E-03 |
| FRMD1 | FERM domain containing 1 | 3.4218 | 3.37E-01 |
| MOB1B | MOB kinase activator 1B | 5.3357 | 1.44E-03 |

ID: hsa03320; Name: PPAR signaling pathway

| Gene Symbol | Gene name | log2FoldChange | p-value |
| --- | --- | --- | --- |
| ME3 | malic enzyme 3 | 1.2004 | 8.67E-01 |
| AQP7 | aquaporin 7 | 1.5540 | 7.81E-03 |
| CPT1A | carnitine palmitoyltransferase 1A | 1.6144 | 3.96E-03 |
| MMP1 | matrix metallopeptidase 1 | 1.8365 | 3.56E-02 |
| FABP3 | fatty acid binding protein 3 | 1.8958 | 9.36E-03 |
| ACSBG1 | acyl-CoA synthetase bubblegum family member 1 | 2.0373 | 2.21E-01 |
| ACOX2 | acyl-CoA oxidase 2 | 3.4303 | 5.81E-02 |

ID: hsa04310; Name: Wnt signaling pathway

| Gene Symbol | Gene name | log2FoldChange | p-value |
| --- | --- | --- | --- |
| MCC | MCC regulator of WNT signaling pathway | 1.1091 | 7.73E-01 |
| CSNK2A1 | casein kinase 2 alpha 1 | 1.2640 | 1.67E-02 |
| TLE3 | TLE family member 3, transcriptional corepressor | 1.3269 | 9.87E-01 |
| ZNRF3 | zinc and ring finger 3 | 1.3428 | 1.87E-02 |
| CTNNB1 | catenin beta 1 | 1.4422 | 3.92E-01 |
| NOTUM | notum, palmitoleoyl-protein carboxylesterase | 1.4698 | 2.85E-02 |
| CTNNBIP1 | catenin beta interacting protein 1 | 1.5760 | 4.18E-01 |
| FRZB | frizzled related protein | 1.7750 | 2.21E-01 |
| SKP1 | S-phase kinase associated protein 1 | 1.8808 | 1.53E-02 |
| DVL2 | dishevelled segment polarity protein 2 | 2.1370 | 1.11E-03 |
| WNT3A | Wnt family member 3A | 2.2460 | 1.91E-02 |
| CCAR2 | cell cycle and apoptosis regulator 2 | 2.3477 | 2.87E-03 |
| RSPO1 | R-spondin 1 | 2.4574 | 1.70E-02 |
| CER1 | cerberus 1, DAN family BMP antagonist | 2.7089 | 2.05E-01 |
| WNT7A | Wnt family member 7A | 3.1154 | 4.02E-02 |
| DKK 2.00 | dickkopf WNT signaling pathway inhibitor 2 | 3.9518 | 2.53E-01 |
| CSNK1A1 | casein kinase 1 alpha 1 | 4.8389 | 1.60E-04 |
| NLK | nemo like kinase | 8.6565 | 9.78E-03 |

ID: hsa04350; Name: TGF-beta signaling pathway

| Gene Symbol | Gene name | log2FoldChange | p-value |
| --- | --- | --- | --- |
| IFNG | interferon gamma | 1.2770 | 6.41E-05 |
| AMH | anti-Mullerian hormone | 1.3459 | 3.01E-03 |
| SKP1 | S-phase kinase associated protein 1 | 1.8808 | 1.53E-02 |
| RPS6KB1 | ribosomal protein S6 kinase B1 | 2.1214 | 1.53E-03 |
| ACVR1C | activin A receptor type 1C | 2.3964 | 2.11E-01 |
| TGIF2 | TGFB induced factor homeobox 2 | 2.9027 | 4.01E-01 |
| PITX2 | paired like homeodomain 2 | 3.2983 | 1.41E-02 |
| RGMA | repulsive guidance molecule BMP co-receptor a | 3.3527 | 1.47E-01 |
| SMURF1 | SMAD specific E3 ubiquitin protein ligase 1 | 4.7181 | 1.30E-01 |
| MAPK3 | mitogen-activated protein kinase 3 | 5.1699 | 1.78E-03 |
| INHBA | inhibin subunit beta A | 6.8088 | 6.49E-04 |

ID: hsa04064; Name: NF-kappa B signaling pathway

| Gene Symbol | Gene name | log2FoldChange | p-value |
| --- | --- | --- | --- |
| LCK | LCK proto-oncogene, Src family tyrosine kinase | 1.0599 | 9.49E-02 |
| CSNK2A1 | casein kinase 2 alpha 1 | 1.2640 | 1.67E-02 |
| CARD14 | caspase recruitment domain family member 14 | 1.2886 | 1.32E-01 |
| SYK | spleen associated tyrosine kinase | 1.4773 | 2.92E-01 |
| MYD88 | MYD88 innate immune signal transduction adaptor | 1.5486 | 2.90E-02 |
| TAB2 | TGF-beta activated kinase 1 (MAP3K7) binding protein 2 | 1.6000 | 8.27E-03 |
| BIRC3 | baculoviral IAP repeat containing 3 | 1.6960 | 1.86E-01 |
| TNFRSF11A | TNF receptor superfamily member 11a | 1.7531 | 4.87E-02 |
| IRAK1 | interleukin 1 receptor associated kinase 1 | 1.8549 | 5.46E-01 |
| ZAP70 | zeta chain of T cell receptor associated protein kinase 70 | 3.3292 | 8.59E-03 |

ID: hsa04550; Name: Signaling pathways regulating pluripotency of stem cells

| Gene Symbol | Gene name | log2FoldChange | p-value |
| --- | --- | --- | --- |
| HOXB1 | homeobox B1 | 1.0173 | 2.55E-02 |
| REST | RE1 silencing transcription factor | 1.2182 | 2.37E-01 |
| CTNNB1 | catenin beta 1 | 1.4422 | 3.92E-01 |
| MAP2K2 | mitogen-activated protein kinase kinase 2 | 1.6937 | 6.10E-01 |
| DVL2 | dishevelled segment polarity protein 2 | 2.1370 | 1.11E-03 |
| WNT3A | Wnt family member 3A | 2.2460 | 1.91E-02 |
| ACVR1C | activin A receptor type 1C | 2.3964 | 2.11E-01 |
| ISL1 | ISL LIM homeobox 1 | 2.8150 | 5.17E-01 |
| MAPK14 | mitogen-activated protein kinase 14 | 3.0573 | 4.55E-01 |
| WNT7A | Wnt family member 7A | 3.1154 | 4.02E-02 |
| LIF | LIF interleukin 6 family cytokine | 5.1172 | 6.63E-02 |
| MAPK3 | mitogen-activated protein kinase 3 | 5.1699 | 1.78E-03 |
| INHBA | inhibin subunit beta A | 6.8088 | 6.49E-04 |

ID: hsa04060; Name: Cytokine-cytokine receptor interaction

| Gene Symbol | Gene name | log2FoldChange | p-value |
| --- | --- | --- | --- |
| IFNG | interferon gamma | 1.2770 | 4.67E-03 |
| AMH | anti-Mullerian hormone | 1.3459 | 4.72E-02 |
| GDF3 | growth differentiation factor 3 | 1.5124 | 3.00E-01 |
| CSF2RB | colony stimulating factor 2 receptor subunit beta | 1.6186 | 1.33E-02 |
| IL7 | interleukin 7 | 1.6276 | 2.27E-01 |
| IFNLR1 | interferon lambda receptor 1 | 1.6560 | 3.53E-03 |
| IFNA7 | interferon alpha 7 | 1.6735 | 1.07E-02 |
| TNFRSF11A | TNF receptor superfamily member 11a | 1.7531 | 4.87E-02 |
| CCL26 | C-C motif chemokine ligand 26 | 1.9090 | 4.70E-01 |
| IL10RA | interleukin 10 receptor subunit alpha | 2.3076 | 2.16E-01 |
| ACVR1C | activin A receptor type 1C | 2.3964 | 2.11E-01 |
| PF4 | platelet factor 4 | 2.6583 | 9.66E-02 |
| LEPR | leptin receptor | 2.7661 | 8.73E-03 |
| CCR4 | C-C motif chemokine receptor 4 | 2.9394 | 3.05E-04 |
| CXCL17 | C-X-C motif chemokine ligand 17 | 3.0373 | 5.89E-05 |
| PF4V1 | platelet factor 4 variant 1 | 3.3533 | 9.66E-02 |
| IL17A | interleukin 17A | 3.7864 | 1.63E-02 |
| PRLR | prolactin receptor | 3.8631 | 1.34E-02 |
| BMP10 | bone morphogenetic protein 10 | 4.0432 | 5.24E-05 |
| GDF15 | growth differentiation factor 15 | 4.0775 | 1.08E-01 |
| IL23A | interleukin 23 subunit alpha | 4.4042 | 7.41E-02 |
| LIF | LIF interleukin 6 family cytokine | 5.1172 | 6.63E-02 |
| IL13 | interleukin 13 | 5.3304 | 1.17E-01 |
| CRLF2 | cytokine receptor like factor 2 | 6.1040 | 3.75E-01 |
| INHBA | inhibin subunit beta A | 6.8088 | 6.49E-04 |

KEGG pathway by WebGestalt gene lists (Downregulate)

ID: hsa04657; Name: IL-17 signaling pathway

| Gene Symbol | Gene name | log2FoldChange | p-value |
| --- | --- | --- | --- |
| ELAVL1 | ELAV like RNA binding protein 1 | -4.1113 | 1.99E-02 |
| TRAF3 | TNF receptor associated factor 3 | -3.2128 | 3.20E-05 |
| TRAF4 | TNF receptor associated factor 4 | -1.6785 | 5.50E-02 |
| IKBKE | inhibitor of nuclear factor kappa B kinase subunit epsilon | -1.5656 | 6.06E-01 |
| CXCL2 | C-X-C motif chemokine ligand 2 | -1.4323 | 2.14E-03 |
| IL6 | interleukin 6 | -1.2320 | 1.01E-02 |
| CXCL3 | C-X-C motif chemokine ligand 3 | -1.2239 | 1.42E-01 |

ID: hsa04350; Name: TGF-beta signaling pathway

| Gene Symbol | Gene name | log2FoldChange | p-value |
| --- | --- | --- | --- |
| ACVR1B | activin A receptor type 1B | -5.5482 | 1.60E-02 |
| PPP2R1B | protein phosphatase 2 scaffold subunit Abeta | -5.1101 | 1.20E-02 |
| HAMP | hepcidin antimicrobial peptide | -3.0634 | 2.65E-03 |
| GREM2 | gremlin 2, DAN family BMP antagonist | -2.8437 | 3.24E-04 |
| AMHR2 | anti-Mullerian hormone receptor type 2 | -1.9205 | 4.72E-02 |
| HFE | homeostatic iron regulator | -1.7470 | 1.54E-04 |
| GDF7 | growth differentiation factor 7 | -1.7268 | 2.21E-01 |
| TFR2 | transferrin receptor 2 | -1.4297 | 3.10E-02 |

ID: hsa04110; Name: Cell cycle

| Gene Symbol | Gene name | log2FoldChange | p-value |
| --- | --- | --- | --- |
| PPP2R1B | protein phosphatase 2 scaffold subunit Abeta | -5.1101 | 1.20E-02 |
| FBXO5 | F-box protein 5 | -4.8305 | 3.73E-02 |
| MAD2L1BP | MAD2L1 binding protein | -3.4155 | 2.71E-03 |
| PPP2R5A | protein phosphatase 2 regulatory subunit B'alpha | -3.2826 | 3.09E-03 |
| ABL1 | ABL proto-oncogene 1, non-receptor tyrosine kinase | -2.6997 | 6.03E-03 |
| BUB3 | BUB3 mitotic checkpoint protein | -2.4499 | 2.24E-02 |
| MTBP | MDM2 binding protein | -2.3171 | 2.42E-01 |
| DDX11 | DEAD/H-box helicase 11 | -1.7064 | 4.71E-03 |
| YWHAE | tyrosine 3-monooxygenase/tryptophan 5-monooxygenase activation protein epsilon | -1.3292 | 1.29E-01 |
| SFN | stratifin | -1.2104 | 9.55E-04 |
| CCNB1 | cyclin B1 | -1.1376 | 1.89E-02 |

ID: hsa04115; Name: p53 signaling pathway

| Gene Symbol | Gene name | log2FoldChange | p-value |
| --- | --- | --- | --- |
| PPM1D | protein phosphatase, Mg2+/Mn2+ dependent 1D | -2.4483 | 3.57E-05 |
| MDM4 | MDM4 regulator of p53 | -2.2426 | 2.24E-02 |
| IGFBP3 | insulin like growth factor binding protein 3 | -1.4314 | 2.31E-02 |
| SFN | stratifin | -1.2104 | 9.55E-04 |
| CCNB1 | cyclin B1 | -1.1376 | 1.89E-02 |

ID: hsa04668; Name: TNF signaling pathway

| Gene Symbol | Gene name | log2FoldChange | p-value |
| --- | --- | --- | --- |
| RIPK3 | receptor interacting serine/threonine kinase 3 | -4.1542 | 9.66E-03 |
| TRAF3 | TNF receptor associated factor 3 | -3.2128 | 1.38E-04 |
| CXCL2 | C-X-C motif chemokine ligand 2 | -1.4323 | 2.14E-03 |
| IL6 | interleukin 6 | -1.2320 | 1.01E-02 |
| CXCL3 | C-X-C motif chemokine ligand 3 | -1.2239 | 1.42E-01 |
| CREB3L2 | cAMP responsive element binding protein 3 like 2 | -1.1341 | 1.74E-02 |
| BCL3 | BCL3 transcription coactivator | -1.0022 | 3.86E-03 |
